# Enhancing Interpretability of Gene Signatures with Prior Biological Knowledge

**DOI:** 10.3390/microarrays5020015

**Published:** 2016-06-08

**Authors:** Margherita Squillario, Matteo Barbieri, Alessandro Verri, Annalisa Barla

**Affiliations:** DIBRIS, University of Genoa, Via Dodecaneso 35, I-16146 Genova, Italy; margherita.squillario@unige.it (M.S.); matteo.barbieri@dibris.unige.it (M.B.); annalisa.barla@unige.it (A.B.)

**Keywords:** gene expression, functional characterization, variable selection, sparse regularization, established domain knowledge, KDVS, Parkinson’s disease, gene ontology

## Abstract

Biological interpretability is a key requirement for the output of microarray data analysis pipelines. The most used pipeline first identifies a gene signature from the acquired measurements and then uses gene enrichment analysis as a tool for functionally characterizing the obtained results. Recently Knowledge Driven Variable Selection (KDVS), an alternative approach which performs both steps at the same time, has been proposed. In this paper, we assess the effectiveness of KDVS against standard approaches on a Parkinson’s Disease (PD) dataset. The presented quantitative analysis is made possible by the construction of a reference list of genes and gene groups associated to PD. Our work shows that KDVS is much more effective than the standard approach in enhancing the interpretability of the obtained results.

## 1. Introduction

Gene expression measures allow for the study of complex diseases such as neurodegenerative diseases and tumors that, unlike Mendelian disorders, depend on the concerted misregulation of several genes. The analysis of microarray data aims at finding a gene signature able to discriminate between groups of samples (e.g., cases and controls, responding or not responding to a specific treatment) and the associated gene functional modules for a pathology of interest. These modules, defined in terms of the established domain knowledge, allow for the assessment of the degree of involvement of the gene signature in relevant pathways, processes, or functions.

**Figure 1:**
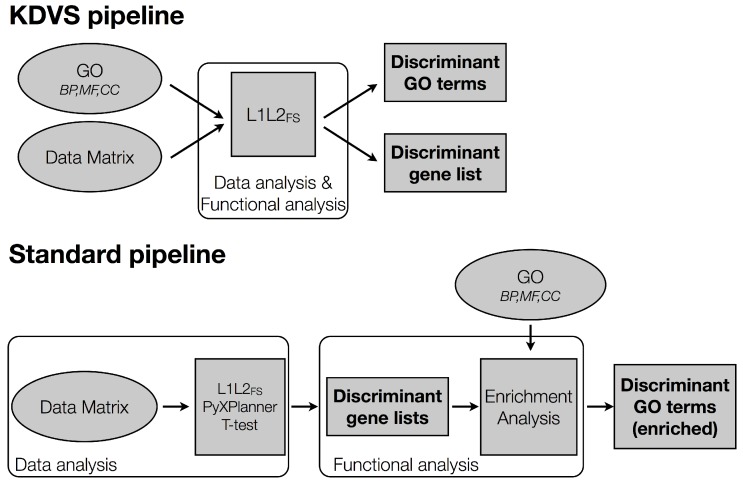
Knowledge Driven Variable Selection (KDVS) and standard pipelines. KDVS embeds the Gene Ontology (GO) domain knowledge into the variable selection step, providing as output a list of discriminant GO terms and genes. The standard pipeline, instead, first selects a gene signature and then performs an enrichment analysis in GO obtaining a discriminant GO term list.

The most common approach to tackle this endeavor, which we refer to as standard pipeline, finds the gene signature and the associated gene functional modules in two steps (see Figure 1). In the first data analysis step, a variable selection method of choice yields a gene signature. In the second step, the obtained signature is functionally characterized by means of an enrichment analysis [1], which aims at recovering biologically relevant genes possibly discarded in the variable selection process. When using Gene Ontology (GO) [2] as the established domain knowledge, the enriched gene modules are GO terms. The obtained results are interpreted by domain experts who evaluate the significance of the selected GO terms by means of the established biological knowledge on the pathology of interest.

Recently, Knowledge Driven Variable Selection (KDVS) [3], an alternative pipeline that uses GO a priori as the established domain knowledge, has been proposed.

The KDVS pipeline (see Figure 1) performs data analysis and functional characterization at the same time, providing, as a final result, a list of GO terms and associated gene signatures relevant for the pathology of interest. This enhances the biological interpretability of the obtained results in terms of functional gene modules.

The aim of this work is to assess quantitatively the effectiveness of KDVS with respect to the *standard pipeline* in the analysis of a gene expression microarray dataset. We restricted our attention to Parkinson’s Disease (PD) as a case study. To this purpose, we built benchmark lists of GO terms and genes by using the Kyoto Encyclopeadia of Genes and Genomes (KEGG) [4], Gene Prospector [5], and the Gene Ontology Annotations (GOA). The obtained benchmark lists allowed us to measure the selection performance in terms of Precision, Recall and F-Measure for both pipelines.

The remainder of this paper is organized as follows. We describe material and methods in Section 2, illustrate the results in Section 3, present our comments in Section 4, and state our final remarks in Section 5. The identified GO terms, genes, and benchmark lists can be found as tables in the Supplementary Material (see Tables S1–S5).

## 2. Experimental Section

In this section, we describe materials and methods of our work. We start with the dataset and the normalization procedure we used, and then we describe the experimental framework, the *standard* and the KDVS pipeline, and the construction of the benchmark lists. Finally, we illustrate the metrics we used to assess performance.

### 2.1. Data and Preprocessing

We devised a binary classification problem of PD cases and controls by using four public microarray datasets stored in the Gene Expression Omnibus (GEO) repository [7]: GSE7621 [8], GSE20292, GSE20291 and GSE20168 [9,10]. All datasets measure the expression on post-mortem brain tissue from patients affected by PD and controls. Specifically, GSE7621 is composed by microarray measures of 16 cases and nine controls deriving from the substantia nigra tissue measured on the HG-U133 Plus 2 platform, characterized by 54,713 probesets. The other three datasets belong to the Superseries GSE20295 and use the HG-U133A platform characterized by 22,283 probesets. GSE20292 is composed by 11 cases and 18 controls from the same brain tissue, the GSE20291 is composed by 15 cases and 20 controls deriving from the putamen brain region, and GSE20168 is composed by 14 cases and 15 controls deriving from the prefrontal area nine brain region.

Normalization of gene expression values was performed on each data matrix using the Robust Multichip Average method [11], with an R script included in the *aroma* package [12]. After normalization, we discarded the control probesets and merged the four preprocessed matrices into one single p×n matrix X, where p=22215 is the number of common probesets and n=118 is the total number of samples (56 cases and 62 controls). An *n*-dimensional vector Y of binary labels distinguishes between cases and controls. In the remainder of the paper, a dataset will be a pair of the type (X,Y).

### 2.2. Methods

#### 2.2.1. Experimental Framework

The statistical analysis of microarray data (like any small set of samples in high-dimensional space) can easily lead to biased results [13]. In order to perform an unbiased analysis, we adopted a two nested cross-validation procedure [14], which we briefly describe here for the sake of completeness. The full dataset (X,Y) is first split in *B* chunks (external split) obtaining *B* datasets $\left(X_{b},Y_{b}\right)$ with b=1,…,B each consisting of B-1 chunks. An optimal model (*i.e.*, a gene signature (actually, a probeset signature) and a classifier) is then obtained for each of the *B* datasets by means of a B-1-fold cross-validation (internal split). Each of the *B* models leads to a possibly different list of selected features; the final aggregate list is obtained by including only those variables appearing in at least a given number of those *B* lists.

#### 2.2.2. The *Standard Pipeline*

The *standard pipeline* reflects the classical approach to extract relevant biological features from normalized high-throughput data sets. It is composed of two steps: *data analysis* and *functional analysis* (Figure 1).

##### Data Analysis

In order to assess the reproducibility of the produced results with the standard pipeline we considered several methods. Fifteen lists of discriminant probesets were obtained by combining three feature selection methods with five classifiers within the unbiased framework described above through the software library PyXPlanner [15]. The three feature selection methods were FilterKBest [16], which selects the top-k features with the highest F-value from a one-way ANOVA test, LASSO [17] and Elastic Net (ENET) [18], which selects the features corresponding to the nonzero components of the vector β minimizing the functional ${∥X\beta -Y∥}_{2}^{2}+\tau {∥\beta ∥}_{1}$ and ${∥X\beta -Y∥}_{2}^{2}+{\alpha \tau ∥\beta ∥}_{1}+\left(1-\alpha \right)\tau {∥\beta ∥}_{2}^{2}$, respectively. The five classification algorithms were *k*-Nearest Neighbors (k-NN), Logistic Regression (LR), Linear Support Vector Machines (LSVM), Ordinary Least Squares (OLS), and Regularized Least Squares (RLS).

A sixteenth list was obtained by means of the univariate method most commonly used in the analysis of this kind of data, the Bonferroni corrected *t*-test.

The last method we used, $\ell_{1}\ell_{2\mathrm{FS}}$, is an embedded regularization method based on ENET, studied in [19,20] and successfully applied in the analysis of high-throughput molecular data [21,22,23,24]. The algorithm, embedded in the unbiased framework of above, is implemented in L1L2Signature [25], a tool in Python based on the L1L2Py [26] and PPlus [27] libraries.

##### Functional Analysis

The functional characterization of the gene signature identified with the standard pipeline was performed through enrichment analysis using the online toolkit WebGestalt [28,29]. WebGestalt takes as input a list of relevant genes/probesets and performs an enrichment analysis based on a hypergeometric test, providing several methods to correct for multiple hypothesis and using several databases (e.g., KEGG or GO) for identifying the most relevant pathways and ontologies in each signature. In other words, given a GO term and a reference set (such as the entire human genome or the list of genes in a microarray platform), the enrichment is based on the comparison between the fraction of signature genes in the GO term and the fraction of GO term genes in the reference set. The signature is enriched in the GO term if the former is larger than the latter fraction.

In our experiments, we enriched each signature using GO, selecting the HG-U133A platform as a reference set, 0.05 as the level of significance, the Bonferroni correction and three as the minimum number of genes in each GO term considered.

#### 2.2.3. The KDVS Pipeline

Let us present the KDVS pipeline of Figure 1. For a more detailed description see [3].

KDVS [30], implemented in Python, is based on the prototype presented in [31]. It uses the established domain knowledge (Gene Ontology release 20100110 [32]) before the actual feature selection step and provides users with a list of discriminant GO terms each coupled with a list of discriminant genes. KDVS consists of three stages: the local integration, knowledge retrieval and post–processing.

The local integration stage accepts the gene expression dataset (X,Y), the microarray annotations (e.g., from GEO), and the representation of biological knowledge (GO). By using the microarray annotation, KDVS builds the mapping from the probeset list to the GO terms and *vice versa* to allow fast querying in both directions. Then, for each GO term *t*, it generates a $p_{s}\times n$ submatrix of gene expression data, with $p_{s}\ll p$, where only the expression values related to genes annotated to t are retained [3]. By construction, the overlap of each pair of submatrices is the same of the corresponding GO terms.

In the knowledge retrieval stage, ${\ell_{1}\ell_{2}}_{FS}$ is performed on each submatrix (GO term), obtaining the classification error as well as the list of selected variables (in our case probesets) that are the most discriminant between the two classes). For all nodes for which $p_{s}<6$, no feature selection is performed.

Finally, the post–processing stage selects the GO terms for which the classification error is below a fixed threshold.

Since KDVS processes one GO domain at a time—Molecular Function (MF), Biological Process (BP) or Cellular Component (CC)—we performed three runs using the same PD dataset. The output, therefore, was obtained by pooling in a single list the three lists of discriminant GO terms as well as the lists of selected probesets.

#### 2.2.4. Benchmark Lists

**Figure 2:**
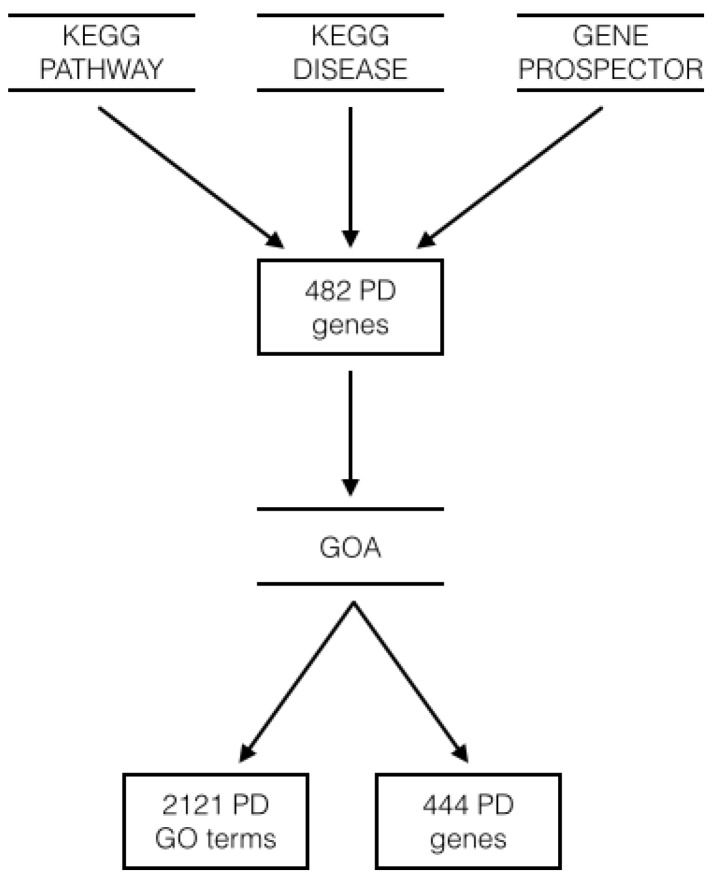
This scheme shows the workflow used to obtain the benchmark gene and GO terms lists. The benchmark gene list is composed of 444 genes and the benchmark GO term list is composed of 2121 terms: 1447 from Biological Process (BP), 446 from Molecular Function (MF) and 228 from Cellular Component (CC).

The benchmark lists were obtained through the workflow depicted in Figure 2.

First, we queried KEGG and Gene Prospector [5]. KEGG is a database of curated biological pathways of the human genome, in addition to other organisms. Gene Prospector, instead, is a tool that allows users to search for genes associated with human diseases, risk factors, and other phenotypes, and may include both experimentally verified and not yet verified biological knowledge. We retrieved genes (1) from the Parkinson’s disease—Homo sapiens pathway of the KEGG PATHWAY database (ID: hsa05012); (2) from the Parkinson’s Disease (PD) entry of the KEGG DISEASE database (ID: H00057); and (3) by querying Gene Prospector for Parkinson’s Disease. The final list contained 482 genes.

Next, by means of Gene Ontology Annotations (GOA) compiled for *Homo sapiens*, we extracted the list of GO terms associated to each of the 482 genes. Evidence codes are provided to motivate each association [6].

Finally, we filtered both lists retaining only the associations based on the following tags: the Experimental Evidence Codes EXP, IDA, IPI, IMP and IGI, IEP, the Traceable Author Statement, and the Inferred by Curator category, which we deemed as the most reliable. In the case of multiple associations between the same gene and GO term we retained the most recent. We obtained benchmark lists of 2121 GO terms (of which 1447 are BP terms, 446 are MF terms and 228 are CC terms) and 444 genes, see Table S1.

#### 2.2.5. Performance Metrics

In customary notation, the true positives (TP) are the benchmark GO terms or genes retained by the pipeline, while the false negatives (FN) are those discarded despite being present in the benchmark. The false positive (FP) are the retained GO terms or genes not in the corresponding benchmark list and the true negatives (TN) those discarded while not in the list.

We evaluated the prediction performance through the mean test error and the Matthews Correlation Coefficient (MCC), which is defined as follows:

$$\mathrm{MCC}=\frac{TP\times TN-FP\times FN}{\sqrt{\left(TP+FP)(TP+FN)(TN+FP)(TN+FN\right)}}.$$

The MCC, unaffected by the presence of unbalanced classes, ranges between −1 and +1. The greater the MCC, the better the prediction with negative score marking below chance performance.

The performance of GO terms and genes selection was measured in terms of Precision, Recall and F-measure with:

$$\text{Precision}=\frac{TP}{TP+FP},\;\text{Recall}=\frac{TP}{TP+FN},\;\text{F-measure}=2\times \frac{\text{Precision}\times \text{Recall}}{\text{Precision}+\text{Recall}}.$$

By definition, Precision, Recall, and F-measure range between 0 and 1, with greater values associated with better performance. High Precision is achieved when the large majority of retained GO terms (or genes) are in the benchmark list, while high Recall is achieved when most of the GO terms (or genes) in the benchmark list are retained. Clearly, by retaining all the terms, it is always possible to obtain perfect Recall at the expense of extremely low Precision values. Therefore, the F-measure, which is high if both Precision and Recall are high, is the score of choice to find the optimal trade-off between Precision and Recall.

For KDVS, we computed Precision and Recall for the cumulative list of GO terms and genes and for each domain separately.

## 3. Results

First, we describe the results obtained with the *standard pipeline*. We divided the dataset in B=9 chunks and performed 8-fold cross-validation. In Table 1, we report the four best test errors obtained from the sixteen methods along with the corresponding MCCs.

The aggregate list of genes for each experiment was obtained by retaining only those that have been selected at least five out of nine times and then enriched according to the procedure described in Section 2.2.2.

We then ran KDVS on the same dataset, using, for each GO term, the same experimental setting: B=9,K=8 and cutoff on gene frequency at 50% (5 out of 9). Based on the test error and standard deviation in Table 1, we decided to retain GO terms associated with a test error less than 31.7%, that is, the ${\ell_{1}\ell_{2}}_{FS}$ mean test error (23.1%) plus its standard deviation (8.6%).

Table S2 and S3 report the list of discriminant GO terms and aggregate list of selected genes for the KDVS pipeline, while Table S4 reports the GO terms and gene lists for the best performing methods of the standard pipeline.

The comparison of the results against the benchmark, in terms of Precision, Recall and F-measure, of KDVS and of the four top performing methods for the standard pipeline is reported in Table 2. We also added the result of the enrichment analysis performed on the list of genes provided by the *t*-test.

**Figure 3:**
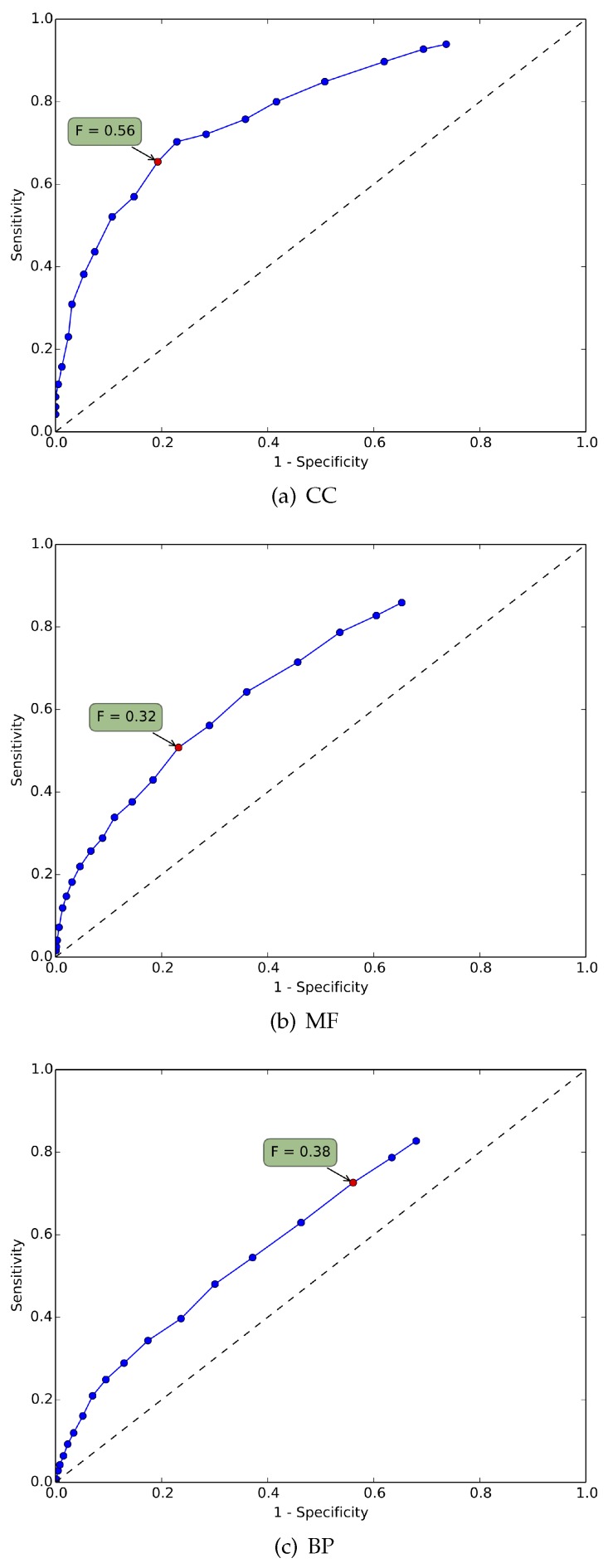
ROC curves for the three GO domains. The plots show the ROC curves (Sensitivity *vs.* 1-Specificity, defined as FP/(TN+FP)) for the KDVS GO terms, for varying values of the threshold error. The highlighted point on the curve is associated with the highest F-measure, reported in the green box.

While in Table 2, the results for KDVS are relative to the three GO domains together, Figure 3 shows three Receiver Operating Characteristic (ROC) curves, one for each domain, where we observe how sensitivity and specificity vary for different values of the error threshold.

## 4. Discussion

### 4.1. Statistical Analysis

Let us first discuss the results illustrated in Table 1. By inspection, we note that the test errors are comparable and well below the chance error (47%). The large values of the standard deviation are likely to be related to the relatively small sample size. Given the complexity of the disease, it is not surprising that the prediction performance of all methods is below 80%. All of the MCC scores indicate a significant correlation between gene expression levels and classes.

As for the results displayed in Table 2, we note that the KDVS pipeline F-measure, for comparable Precision values, is between 40 and 200 times greater than the F-measures obtained with the *standard pipeline*. Interestingly, the performance of the standard pipeline does not change much with the variable selection method (including the widely used *t*-test). Since the best performance of the standard pipeline is obtained by means of the $\ell_{1}\ell_{2FS}$ feature selection method, we conclude that the actual gain of KDVS, which uses $\ell_{1}\ell_{2FS}$ as the variable selection engine, is about 40-fold.

Let us comment on the results in terms of absolute figures instead of percentages. In Table S5, we listed the TPs for all the methods in Table 2. While the TPs for KDVS are 270, the number of TPs for each of the five methods of the standard pipeline range from one to five. All in all, of the seven different GO terms collectively identified by the five methods, four are also in KDVS list, and two are direct ancestors of two KDVS GO terms. Clearly, in order to be profitably explored by domain experts, the KDVS list needs to be refined. On the other hand, the variability of the GO terms returned by the standard pipeline questions the reliability of the produced results.

It is also interesting to consider the results in Table 2 from the gene point of view. In the standard pipeline, the gene enrichment produces a GO term list starting from a gene list. Not surprisingly, for all methods in the standard pipeline, the GO term F-measure is significantly smaller than the corresponding genes F-measure, while the opposite holds for KDVS, consistently with the underlying concept.

Finally, the ROC curves in Figure 3 show that the considerable edge of KDVS *vs.* the *standard pipeline* remains true in each of the three GO domains considered separately.

### 4.2. Biological Significance

Here, we comment on the results of the KDVS pipeline from a biological viewpoint. From the ROC curves shown in Figure 3, we note that the CC domain terms yield a better performance than MF and BP terms with respect to both specificity and sensitivity. By construction, the benchmark list may contain GO terms with broad meaning. The thorough review for each GO domain presented in the remainder of this Section shows that the the biological features of the selected GO terms common to the benchmark (see Table S1) are often relevant for a neurodegenerative disease such as PD.

For the CC domain, the overlap consist of 69 terms, mainly related to: (i) mitochondrion (e.g., matrix, crista, outer and inner cellular membranes, mitochondrial respiratory chain, mitochondrial proton–transporting ATP synthase complex); (ii) neurons (e.g., synapse, synaptic vescicle, axon, dendrite and dendritic shaft); (iii) various cell regions like cell-cell junctions, proteinaceous extracellular matrix, cell cortex, filopodium, actin and microtubule cytoskeleton; and (iv) cytoplasmatic vescicles and several organelles such as the nucleus, endoplasmatic reticulum, Golgi, centrosomes and lysosomes.

For the MF domain, the overlap consists of 71 terms, mainly related to: (i) binding of motor proteins; (ii) ions and groups (*i.e.*, zinc, calcium, magnesium, iron manganese, copper, sodium, potassium, ATP, GTP); (iii) nucleotidic acids (*i.e.*, chromatin, single- and double-stranded DNA, mRNA); (iv) integrins, signaling proteins, low-density lipoproteins, tyrosine kinase; (v) specific proteins or proteins categories like polyubiquitin, apoliprotein E, dopamine, heat shock proteins, NF-kappaB, protein N and *C*-terminus, SH3 domains, piridoxal phosphate, phosphatidylinositol; and (vi) unfolded proteins. The molecular functions related to the selected GO terms involve enzymes (e.g., hydrolase, peptidase, especially serine and cysteine-type peptidase), calcium channel, small conjugating protein ligase ubiquitin, cytochrome-c oxidase, NADH dehydrogenase and ubiquinol-cytochrome-c reductase.

For the BP domain, the overlap consists of 130 terms, mainly related to: (i) various kind of metabolic processes concerning lipids, carbohydrates (e.g., glycogen), ATP or dopamine; (ii) development of the central nervous system, the forebrain, the heart and the skeletal tissue; and (iii) defense response, in particular from unfolded proteins and from viruses that prompt the differentiation of B cells, and from inflammation (*i.e.*, acute-phase), oxidative stress, hypoxia, DNA damage, heat and tumor necrosis factors. The BP terms control cell adhesion, differentiation (*i.e.*, B and myeloid), migration, signaling, cell cycle arrest, respiration, growth, differentiation and proliferation. The involved pathways concern Notch receptors, which regulate cell–cell communication in several ways (acting, in particular, in the central nervous system and in the heart) and the nerve growth factors, fundamental for the growth, maintenance, and survival of neurons. The involvement of the mitochondrion is essential as confirmed by the GO terms: mitochondrial electron transport, NADH to ubiquinone and regulation of mitochondrial membrane potential. Among the regulation processes related to PD, it is important to underline neurone differentiation, the positive regulation of anti-apoptosis, and the negative regulation of axonogenesis and of locomotion.

## 5. Conclusions

The main aim of this work was to assess the effectiveness of the KDVS pipeline with respect to the standard pipeline for the analysis of microarray data. While the standard pipeline first selects the relevant variables and then uses the established biological domain knowledge to reconstruct relevant functional modules, KDVS obtains relevant functional modules by embedding the domain knowledge in the variable selection process.

We considered PD as a case study and constructed lists of GO terms and genes, obtained by means of the available PD knowledge, which we use as benchmark. Our analysis shows that, for comparable values of precision, the recall and F-measure of KDVS are significantly higher (about two orders of magnitude) than the standard pipeline. Furthermore, KDVS, providing GO terms as output, enhances the biological interpretability suggesting an explanation of the phenomenon under study in terms of functional gene modules rather than single molecular variables. On the basis of the obtained results, we believe that the proposed approach can be regarded as a first step toward the construction of a data and knowledge driven process for the discovery of novel associations.

## Figures and Tables

**Table 1:** Top performing methods for the *standard pipeline*. For each method, the average test error, standard deviation (SD), and MCC are reported.

Experiment	Test Error ± SD (%)	MCC
${\ell_{1}\ell_{2}}_{FS}$	23.1 ± 8.6	0.54
**FiltKBest & LR**	22.0 ± 9.7	0.56
**LASSO & LR**	22.0 ± 8.2	0.56
**ENET & LR**	24.6 ± 7.1	0.51

**Table 2:** Selection performance of Knowledge Driven Variable Selection (KDVS) and five different instances of the standard pipeline *vs.* the benchmark. Precision, Recall and F-measure are reported for KDVS, the best four methods of Table 1 and the *t*-test for GO terms and genes.

	GO Terms			Genes		
Experiments	Precision (%)	Recall (%)	F-measure ($\times 10^{-3}$)	Precision (%)	Recall (%)	F-measure ($\times 10^{-3}$)
**KDVS all domains**	44.0	12.7	**197.4**	7.5	25.5	**115.5**
${\ell_{1}\ell_{2}}_{FS}$	71.4	0.2	4.8	10.4	1.1	20.4
**FiltKBest & LR**	50.0	0.1	1.0	3.5	0.5	8.0
**LASSO & LR**	50.0	0.1	2.8	18.8	0.7	13.1
**ENET & LR**	62.5	0.2	4.8	16.7	0.9	17.1
***t*-test**	50.0	0.1	1.0	2.5	0.2	4.2

## Supplementary Materials

The following are available online at http://www.mdpi.com/2076-3905/5/2/15/s1. Table S1: Benchmark GO terms and genes, Table S2: GO terms selected by KDVS, Table S3: Genes selected by KDVS, Table S4: GO terms and genes selected by the standard pipeline, Table S5: True positives.

## Abbreviations

PD: Parkinson’s Disease
GEO: Gene Expression Omnibus
KDVS: Knowledge Driven Variable Selection
${\ell_{1}\ell_{2}}_{FS}$: $\ell_{1}\ell_{2}$ feature selection framework
GO: Gene Ontology
FiltKBest: Filter K-Best selection based on ANOVA
LASSO: Lasso
ENET: Elastic Net
KNN: K Nearest Neighbor
LR: Logistic Regression
LSVM: Linear Support Vector Machine
OLS: Ordinary Least Square
RLS: regularized least square
DAG: Directed Acyclic Graph
MF: Molecular Function
BP: Biological Process
CC: Cellular Component
KEGG: Kyoto Encyclopaedia of Genes and Genomes
GOA: Gene Ontology Annotations
TAS: Traceable Author Statement
IC: Inferred by Curator
TP: True Positive
TN: True Negative
FP: False Positive
FN: False Negative
